# Newcastle disease virus-vectored West Nile fever vaccine is immunogenic in mammals and poultry

**DOI:** 10.1186/s12985-016-0568-5

**Published:** 2016-06-24

**Authors:** Jinliang Wang, Jie Yang, Jinying Ge, Ronghong Hua, Renqiang Liu, Xiaofeng Li, Xijun Wang, Yu Shao, Encheng Sun, Donglai Wu, Chengfeng Qin, Zhiyuan Wen, Zhigao Bu

**Affiliations:** State Key Laboratory of Veterinary Biotechnology, Harbin Veterinary Research Institute, Chinese Academy of Agricultural Sciences, 427 Maduan Street, Harbin, Heilongjiang 150001 People’s Republic of China; Department of Virology, State Key Laboratory of Pathogens and Biosecurity, Beijing Institute of Microbiology and Epidemiology, Beijing, China

**Keywords:** West Nile fever, Newcastle disease virus vectored vaccine, Neutralizing antibody, T cell response, Mammal, Poultry

## Abstract

**Background:**

West Nile virus (WNV) is an emerging zoonotic pathogen which is harmful to human and animal health. Effective vaccination in susceptible hosts should protect against WNV infection and significantly reduce viral transmission between animals and from animals to humans. A versatile vaccine suitable for different species that can be delivered via flexible routes remains an essential unmet medical need. In this study, we developed a recombinant avirulent Newcastle disease virus (NDV) LaSota strain expressing WNV premembrane/envelope (PrM/E) proteins (designated rLa-WNV-PrM/E) and evaluated its immunogenicity in mice, horses, chickens, ducks and geese.

**Results:**

Mouse immunization experiments disclosed that rLa-WNV-PrM/E induces significant levels of WNV-neutralizing antibodies and E protein-specific CD4+ and CD8+ T-cell responses. Moreover, recombinant rLa-WNV-PrM/E elicited significant levels of WNV-specific IgG in horses upon delivery via intramuscular immunization, and in chickens, ducks and geese via intramuscular, oral or intranasal immunization.

**Conclusions:**

Our results collectively support the utility of rLa-WNV-PrM/E as a promising WNV veterinary vaccine candidate for mammals and poultry.

## Background

West Nile virus (WNV) is the causative agent of West Nile fever (WNF), a major emerging zoonotic disease shown to have a significant negative impact on both human and animal health since the first recorded case in Uganda in 1937. WNV is a member of the genus *Flavivirus* belonging to the family *Flaviviridae*. The virus is one of the most widespread arthropod-transmitted pathogens, and is extensively distributed worldwide throughout Africa, Europe, Asia and North America. WNV has a broad host spectrum comprising several species of birds (including poultry), mammals, amphibians and reptiles. *Culex* mosquitoes play an important role as the primary global WNV transmission vector, and are responsible for the incidental infection of humans and horses, which are considered dead-end hosts of WNV [1–4].

Vaccination in sensitive host animals, especially those abundant in number and closely associated with humans, such as horses, poultry and other bird species, should protect against WNV infection and significantly reduce transmission between animals and from animals to humans. Currently, several injection-delivered vaccines [5–8] are licensed for horses, but not other sensitive host animals. A versatile vaccine suitable for different species that can be delivered via flexible administration routes therefore remains an unmet medical requirement.

Newcastle disease virus (NDV) has been actively developed and evaluated as a vaccine vector for the control of human and animal diseases [9–16]. NDV vector vaccines can be effectively delivered via intramuscular or intratracheal inoculation in mammals and intramuscular, intranasal or oral (through water or feed) inoculation in poultry [11, 12, 17–21]. In the current study, we generated a recombinant nonvirulent NDV LaSota virus strain expressing WNV pre-membrane (PrM) and envelope protein (E), two surface glycoproteins that form a heterodimer on the viral surface [22] and are responsible for eliciting the majority of protective immune responses [23]. Immunogenicity of the recombinant NDV in mammals and poultry delivered via different immunization routes was further evaluated.

## Methods

### Construction of recombinant NDV LaSota virus

The chemically synthesized mammalian codon-optimized WNV *PrM/E* gene (strain NY99, GenBank No. DQ211652.1) was cloned and inserted into the *Pme* I site between the *P* and *M* genes of full-length genomic cDNA of NDV LaSota [11]. The resultant plasmid was co-transfected with eukaryotic plasmids expressing NDV nucleoprotein (NP), phosphate protein (P) and large polymerase protein (L), following an established protocol [11]. The rescued recombinant virus was designated rLa-WNV-PrM/E. Expression of WNV PrM and E proteins was confirmed via indirect immunofluorescence and western blot assays. Mouse anti-WNV E monoclonal antibody (developed in our laboratory), mouse anti-PrM monoclonal antibody [24] and chicken anti-NDV serum [11] was used as primary antibodies. Fluorescein isothiocyanate (FITC)-conjugated goat anti-mouse antibody (Sigma, St. Louis, MO) and Tetramethylrhodamine (TRITC)-conjugated rabbit anti-chicken antibody (Sigma, St. Louis, MO) was used as secondary antibodies for immunofluorescence assay. Chicken anti-NDV serum and mouse anti-WNV serum (developed in our laboratory) were used as primary antibodies, horseradish-peroxidase (HRP)-conjugated goat anti-chicken IgG and goat anti-mouse IgG (SouthernBiotech, Birmingham, AL) were used as secondary antibodies for western blot assay.

To determine the pathogenicity of rLa-WNV-PrM/E in poultry, mean death time, intracerebral pathogenicity index, and intravenous pathogenicity index were determined in embryonated specific pathogen-free (SPF) chickens or eggs according to the OIE Manual [25]. To assess pathogenicity in mouse, ten 6-week-old female C57BL/6 mice (Vital River, Beijing, China) were inoculated intramuscularly with 0.1 ml diluted allantoic fluid containing 1 × 10^8^ EID_50_ (50 % Embryo Infectious Dose) rLa-WNV-PrM/E and intranasally with 0.03 ml diluted allantoic fluid containing 3 × 10^7^ EID_50_ rLa-WNV-PrM/E. Mice were examined daily for 3 weeks for signs of illness, weight loss or death.

### Animal immunization studies

For mouse immunization, ten 6-week-old female C57BL/6 mice (Vital River, Beijing, China) were intramuscularly vaccinated with 0.1 ml diluted allantoic fluid containing 1 × 10^8^ EID_50_ rLa-WNV-PrM/E twice with a 3-week interval. Splenocytes for assay of E protein-specific CD4+ and CD8+ T-cell responses were harvested 10 days after the first or second dose. Serum samples for the serological assay were prepared 2 weeks after each dose.

For horse immunization, five adult horses were intramuscularly inoculated with 2 ml diluted allantoic fluid containing 2 × 10^9^ EID_50_ rLa-WNV-PrM/E, and five administered with 2 ml phosphate-buffered saline (PBS) as the control group. Three weeks after the first dose, a booster with the same vaccine was delivered using the same dosage and route. Serum samples were collected for serological assay 2 weeks after each immunization.

For poultry immunization, three groups (ten per group) of 4-week-old SPF chickens were assessed: intramuscular inoculation with 0.1 ml diluted allantoic fluid containing 1 × 10^8^ EID_50_ rLa-WNV-PrM/E (Group One), oral inoculation with 10 ml diluted allantoic fluid containing 1 × 10^10^ EID_50_ rLa-WNV-PrM/E mixed with 500 g chicken feed and 300 ml water (Group Two), whereby feeding was stopped 5 h before inoculation, and intramuscular and oral inoculation with PBS (Group Three). Three groups (ten per group) of 4-week-old SPF ducks were immunized following the above procedure. For immunization of geese, four groups (15 per group) of 4-week-old birds were examined: intramuscular inoculation with 0.5 ml diluted allantoic fluid containing 5 × 10^8^ EID_50_ rLa-WNV-PrM/E (Group One), intranasal inoculation with 0.5 ml diluted allantoic fluid containing 5 × 10^8^ EID_50_ rLa-WNV-PrM/E via eye drops and nostril instillation (Group Two), oral inoculation with 0.5 ml diluted allantoic fluid containing 5 × 10^8^ EID_50_ rLa-WNV-PrM/E via buccal cavity instillation (Group Three), and intramuscular inoculation with 0.5 ml PBS (Group Four). Three weeks after the first dose, chickens, ducks and geese were boosted with the vaccine using the same doses and routes. Serum samples were collected for serological assay 2 weeks after each immunization. All poultry were housed in the Experimental Animal Center of Harbin Veterinary Research Institute.

### Analysis of WNV-specific IgG, neutralizing and NDV HI antibodies

Enzyme-linked immunosorbent assay (ELISA) for determining antigen-specific IgG in mouse serum was performed as described previously [26]. Briefly, purified mammalian cells producing WNV virus-like particles (4 μg/ml, containing PrM and E proteins, unpublished) were used as coating antigen. Antibodies were detected using HRP-labeled goat anti-mouse IgG (SouthernBiotech, Birmingham, AL) secondary antibody. A standard curve was generated by coating with serially diluted mouse IgG (Southern Biotech, Birmingham, AL) at known concentrations. A linear equation was obtained based on the standard IgG concentration and their O.D values, thus the concentration of WNV-specific IgG was calculated according to the linear equation based on their O.D values and expressed as the amount of IgG per ml of serum (ng/ml). The above coating antigen was also used for ELISA detection of WNV-specific IgG in horse and poultry sera. HRP-labeled goat anti-horse IgG (SouthernBiotech, Birmingham, AL) and goat anti-chicken IgG (SouthernBiotech, Birmingham, AL) were used as secondary antibodies for horse and chicken serum detection, and mouse anti-duck IgG (AbD Serotec, Oxford, UK) and HRP-labeled goat anti-mouse IgG (SouthernBiotech, Birmingham, AL) for duck and goose serum detection. Due to the lack of purified IgG for these animals, results were expressed as O.D. values relative to negative controls.

Mouse serum neutralizing antibody levels were determined using the WNV plaque reduction neutralization test (PRNT) in the Biosafety Level 3 facility of Beijing Institute of Microbiology and Epidemiology. Briefly, 320 μl of 10-fold serially diluted mouse serum (heat-inactivated at 56 °C for 30 min before use) was mixed with 320 μl medium containing 150 plaque-forming units (PFU) of WNV (strain NY99) and incubated at 37 °C for 1 h. Next, the mixture was added to BHK-21 cells in the wells of a six-well plate and incubated at 37 °C for 1 h. Following removal of the mixture, cells washed three times with PBS. Cells were overlaid with 2 ml DMEM-agarose, and incubation continued at 37 °C. After 72 h, cells were fixed with 4 % paraformaldehyde and subsequently stained with 1.5 % crystal violet to visualize plaques. Neutralization titers were expressed as the reciprocal of the highest dilution of serum showing at least 50 % reduction in number of plaques, compared with the negative control. Neutralizing antibodies of chicken, duck, goose and horse sera were not assessed due to unavailability of the BSL-3 facility during the experimental period. NDV hemagglutinin inhibition (HI) antibodies of immunized animals were determined following a previously described protocol [11].

### Flow cytometric analysis of the mouse CD4+ and CD8+ T-cell response

The WNV E protein-specific CD4+ and CD8+ T-cell response in C57BL/6 mice was determined via flow cytometry using established protocols [27]. rLa-WNV-PrM/E-immunized mice were sacrificed on day 10 after the first and second immunizations. Mouse splenocytes were prepared as documented by Ye et al. [28]. Briefly, spleens were removed from euthanized mice, cut into small sections, and homogenized by gentle rubbing. After low-speed centrifugation, the supernatant was removed, cells gently re-suspended in red blood cell lysis buffer (Sigma), and incubated on ice for 1 min. Splenocytes (1 × 10^6^) were stimulated with 20 μg/ml WNV E-specific CD4 peptide (PVGRLVTVNPFVSVA, H-2^*b*^, [29]) or CD8 peptide (LGMSNRDFL, H-2D^*b*^, [30] for 6 h in presence of 10 ng/ml Brefeldin A (eBioscience, San Diego, CA) to assess the CD4+ and CD8+ T-cell response, respectively. Cells were washed twice with PBS containing 3 % fetal calf serum and subsequently stained with Peridinin-Chlorophyll-Protein-Complex (PerCP)-conjugated rat anti-mouse CD4 (or CD8) and phycoerythrin (PE)-conjugated rat anti-mouse CD3 antibody (BD Pharmingen, San Diego, CA). Next, cells were fixed and permeabilized with Fix&Perm Buffer (eBioscience, San Diego, CA) and stained for intracellular interferon-gamma (IFN-γ) with an allophycocyanin (APC)-conjugated rat anti-mouse IFN-γ antibody (BD Pharmingen). The levels of CD4+ or CD8+ T-cell responses were determined using flow cytometry on a BD FACSAria Station (BD Immunocytometry Systems, San Jose, CA). Data were analyzed with FlowJo software (Treestar Inc, Ashland, OR).

### Statistical analysis

Data on virus titers, antibody titers and mouse T cell responses were analyzed using two-tailed Student’s *t* test with the Excel program (Microsoft, Redmond, WA). To describe the *p* value significance, the following convention was used: not significant, *p* > 0.05; significant, *p* ≤ 0.05; highly significant, *p* ≤ 0.01.

## Results

### Generation of rLa-WNV-PrM/E virus and in vitro characterization

**Fig. 1:**
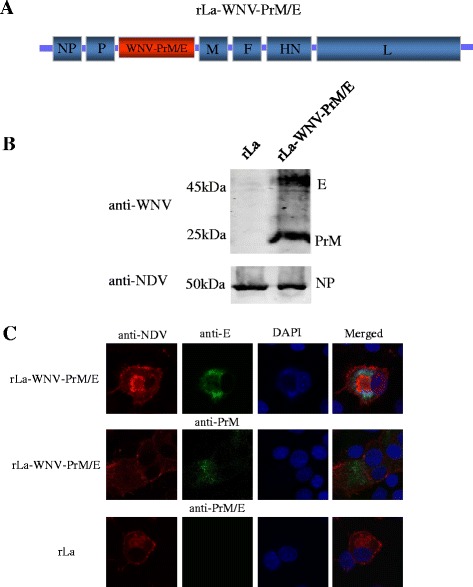
Generation of recombinant NDV expressing WNV PrM/E. Schematic representation of the rLa genome with the *Pme I* restriction site introduced between the *P* and *M* genes for WNV PrM/E gene insertion (**a**). Western blot (**b**) and immunofluorescence staining for detection of WNV PrM/E expression in rLa-WNV-PrM/E- infected BHK-21 cells (**c**)

Recombinant NDV expressing WNV PrM/E proteins was generated by inserting the *PrM/E* gene between the *P* and *M* genes in NDV genome cDNA (Fig. 1a). The presence of PrM/E was confirmed via RT-PCR. PrM/E protein expression was confirmed via western blot and indirect immunofluorescence staining of rLa-WNV-PrM/E-infected BHK-21 cells. Western blot detected the presence of both E (~45 kDa) and PrM (~25 kDa) proteins (Fig. 1b), which were further confirmed via indirect immunofluorescence with specific monoclonal antibodies against each protein. As expected, rLa-infected BHK-21 cells were not stained (Fig. 1c).

The growth titer of rLa-WNV-PrM/E in embryonated chicken eggs was comparable to that of parental rLaSota. Genetic stability of rLa-WNV-PrM/E was assessed by serial passage of the virus in SPF chicken eggs, and confirmed with RT-PCR and immunofluorescence (data not shown). Mean death time (>120 h), intracerebral pathogenicity index (=0), and intravenous pathogenicity index (=0) results demonstrated the lentogenic nature of rLa-WNV-PrM/E in poultry (data not shown). The genetic stability of WNV PrM/E gene within rLa-WNV-PrM/E was assessed by serially passage (at least 10 passages) of the virus in embryonated SPF chicken eggs, the presence and expression of PrM/E was confirmed by RT-PCR and indirect immunofluorescence assay. The results demonstrated the PrM/E gene can be stably maintained and expressed. Ten mice receiving intramuscular inoculation at a dose of 1 × 10^8^ EID_50_ and intranasal inoculation of 3 × 10^7^ EID_50_ rLa-WNV-PrM/E survived with no abnormalities during the 2-week observation period. No significant differences in weight gain were observed after inoculation. Our results indicate rLa-WNV-PrM/E is safe for mice (data not shown).

### The recombinant virus induces significant WNV-specific humoral and T-cell responses in mice

**Fig. 2:**
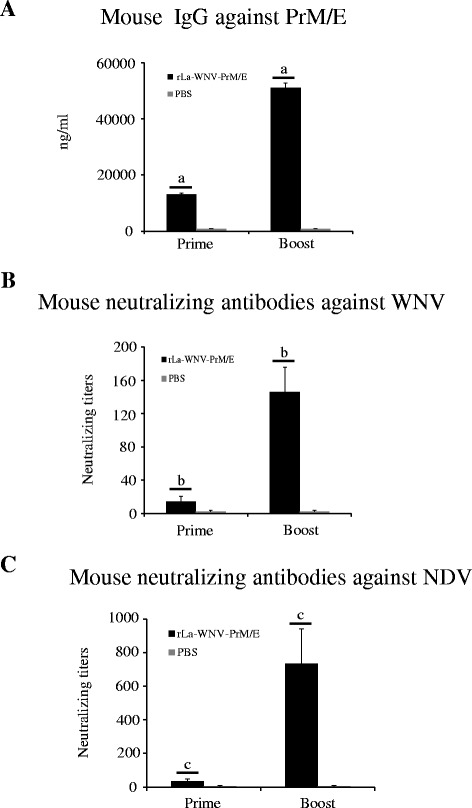
Humoral responses in mice. Mice were intramuscularly inoculated with two doses of rLa-WNV-PrM/E with a 3-week interval. Serum ELISA antibody to WNV E (**a**), neutralizing antibody against WNV (**b**) and neutralizing antibody to NDV (**c**) were assessed at different times post-inoculation. Data are presented as mean ± SD for each group. (a), (b), and (c): *p <* 0.01, significance of the differences in antibody amounts between the first and second dose

WNV-specific IgG (Fig. 2a) was detected using ELISA. Notably, the IgG antibody level was significantly boosted after the second dose (*p* < 0.01). Serum neutralizing antibodies were analyzed with a WNV plaque reduction assay*.* As shown in Fig. 2b, WNV-neutralizing antibodies were detected after the first dose, and significantly boosted after the second dose (*p* < 0.01). NDV neutralizing antibodies were also detected after the first dose, and significantly boosted after the second dose (*p* < 0.01) (Fig. 2c).

**Fig. 3:**
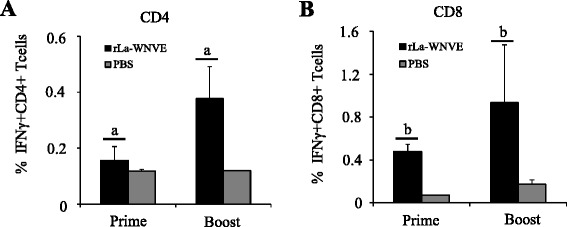
T-cell responses in mice. Mice were sacrificed at the tenth day post-immunization, and their splenocytes were prepared and stimulated with E protein CD4 or CD8 epitope peptide. Cells were stained for cell surface CD4 or CD8 as well as intracellular IFN-γ protein before flow cytometry analysis. Statistical results (percentages) of E protein- specific IFN-γ-producing CD4+ T cells (**a**) and CD8+ T cells (**b**) are shown. Data are presented as mean ± SD of five mice for each group. (a), (b): *p* < 0.05, significance of the differences in values between the first and second dose

The WNV E protein-specific CD4 epitope (PVGRLVTVNPFVSVA, H-2^*b*^, [29] and CD8 epitope (LGMSNRDFL, H-2D^*b*^, [30] were used to assess rLa-WNV-PrM/E-induced specific T-cell responses in mice. As shown in Fig. 3, rLa-WNV-PrM/E immunization induced significant epitope-specific IFN-γ-producing CD4+ and CD8+ T-cell responses after the first dose, which were significantly boosted after the second dose (*p* < 0.05).

### rLa-WNV-PrM/E administered via different immunization routes induces significant WNV IgG antibody production in horses and poultry

**Fig. 4:**
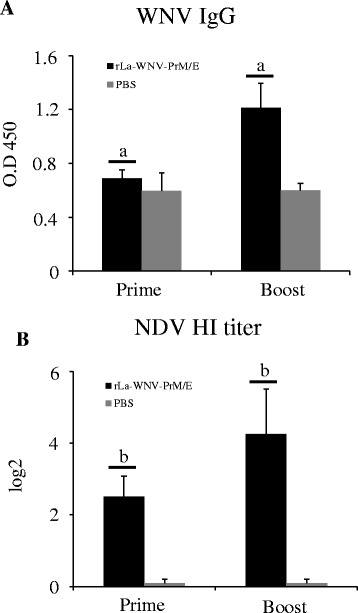
WNV IgG and NDV HI antibody titers of horses. Horses were intramuscularly inoculated with two doses of rLa-WNV-PrM/E with a 3-week interval. Blood samples were collected 2 weeks after the first dose (prime) and the second dose (boost) for antibody assays. Levels of the specific ELISA antibody (**a**) and vector (NDV)-specific HI antibody were determined (**b**). Horse serum was 1000× diluted for ELISA. Data are presented as mean ± SD for each group. (a): *p* < 0.01, (b): *p <* 0.05, significance of the differences in antibody amounts between the first and second dose of immunization

**Fig. 5:**
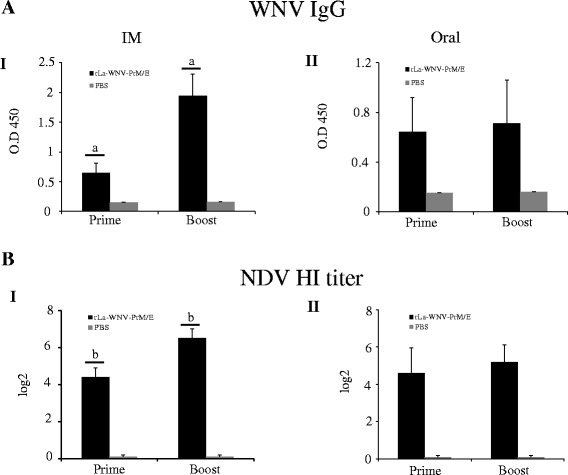
WNV IgG and NDV HI antibody titers of chickens. SPF chickens were intramuscularly or orally inoculated with two doses of rLa-WNV-PrM/E with a 3-week interval. Blood samples were collected 2 weeks after the first dose (prime) and second dose (boost) for antibody assays. Levels of the specific ELISA antibody (**a**) and vector (NDV)-specific HI antibody were determined (**b**). Chicken serum was 3000× diluted for ELISA. Data are presented as mean ± SD for each group. (a), (b): *p <* 0.01, significance of the differences in antibody amounts between the first and second dose of immunization

**Fig. 6:**
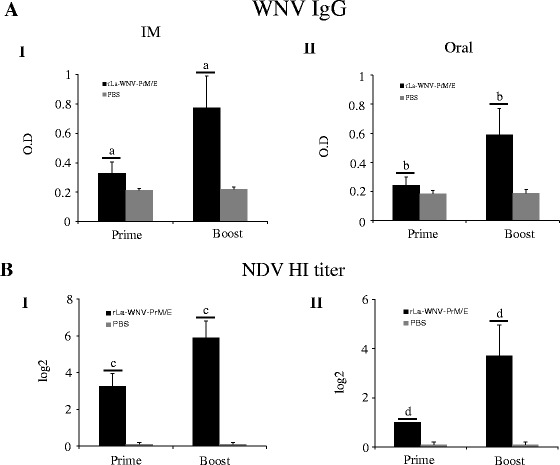
WNV IgG and NDV HI antibody titers of ducks. SPF ducks were intramuscularly or orally inoculated with two doses of rLa-WNV-PrM/E with a 3-week interval. Blood samples were collected 2 weeks after the first dose (prime) and second dose (boost) for antibody assays. Levels of the specific ELISA antibody (**a**) and vector (NDV)-specific HI antibody were determined (**b**). Duck serum was 1000× diluted for ELISA. Data are presented as mean ± SD for each group. (a), (b), (c), (d): *p <* 0.01, significance of the differences in antibody amounts between the first and second dose of immunization

**Fig. 7:**
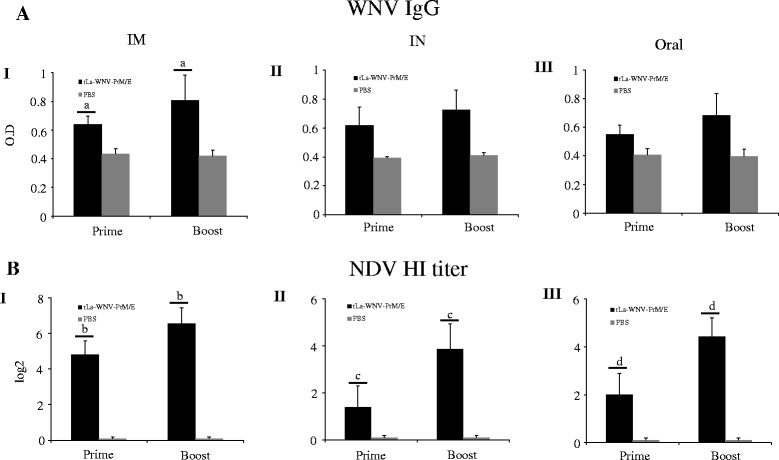
WNV IgG and NDV HI antibody titers of geese. Domestic geese were intramuscularly, intranasally or orally inoculated with two doses of rLa-WNV-PrM/E with a 3-week interval. Blood samples were collected 2 weeks after the first dose (prime) and second dose (boost) for antibody assays. Levels of the specific ELISA antibody (**a**) and vector (NDV)-specific HI antibody were determined (**b**). Goose serum was 1000× diluted for ELISA. Data are presented as mean ± SD for each group. (a): *p* < 0.05, (b), (c), (d): *p <* 0.01, significance of the differences in antibody amounts between the first and second dose of immunization

Given that rLa-WNV-PrM/E induces good humoral responses in mice, we performed horse immunization with the vaccine. Horses received two doses of the vaccine via the intramuscular route with a 3-week interval. WNV-specific IgG was detected after the first dose, and significantly boosted after the second dose (*p* < 0.01) (Fig. 4a). HI antibodies against NDV were also detected in horse after the first dose, and significantly boosted after the second dose (*p* < 0.01) (Fig. 4b). To determine whether rLa-WNV-PrM/E induces an immune response in poultry, SPF chickens were intramuscularly or orally inoculated with the recombinant virus twice with a 3-week interval. In intramuscularly immunized chickens, the WNV-specific IgG was detected after the first dose, and significantly boosted after the second dose (*p* < 0.05) (Fig. 5a I). In orally immunized chickens, IgG was also detected after the first dose, but only slightly boosted after the second dose (Fig. 5a II). NDV HI antibodies was detected after the first dose, and significantly boosted after the second dose (*p* < 0.05) in intramuscularly immunized chickens (Fig. 5b I). In orally immunized chickens, NDV HI antibody was also detected after the first dose, but only slightly boosted after the second dose with no statistical significance (Fig. 5b II). The same immunization procedure was performed for ducks. WNV IgG in intramuscularly (Fig. 6a I) and orally (Fig. 6a II) immunized duck sera was induced after the first dose, and boosted significantly after the second dose (*p* < 0.05). NDV HI antibodies were detected after the first dose, and significantly boosted after the second dose in intramuscularly (Fig. 6b I) and orally immunized ducks (*p* < 0.01) (Fig. 6b II). Groups of outbred geese were either intramuscularly, intranasally or orally inoculated with rLa-WNV-PrM/E. Intramuscularly immunized geese produced detectable WNV IgG after the first dose, which was significantly boosted after the second dose (*p* < 0.05) (Fig. 7a I). Intranasally (Fig. 7a II) and orally (Fig. 7a III) immunized geese also produced a detectable level of IgG after the first dose, which was only slightly boosted after the second dose (*p* > 0.05). NDV HI antibodies were detected after the first dose, and significantly boosted after the second dose in intramuscularly (Fig. 7b I), intranasally (Fig. 7b II) and orally (Fig. 7b III) immunized geese (*p* < 0.01).

## Discussion

WNV is an important zoonotic pathogen widely distributed geographically, with emergence of increasingly neuroevasive strains. Here, a recombinant NDV LaSota virus expressing WNV PrM and E proteins, rLa-WNV-PrM/E, was constructed as a candidate veterinary vaccine for WNV prevention and control. rLa-WNV-PrM/E elicited significant levels of neutralizing antibodies and WNV-specific T-cell responses in mice, as well as WNV-specific IgG in horses, chickens, ducks, and geese, support the immunogenicity of the newly generated recombinant virus in mammals and poultry.

Mice are sensitive to WNV infection, and thus commonly used as the model animal for WNV vaccine evaluation and other related studies. In our experiments, mice intramuscularly inoculated with rLa-WNV-PrM/E produced significant WNV-neutralizing antibodies and specific IgG. Neutralizing antibodies play a crucial role in WNV control and clearance [31]. We used the 50 % plaque reduction assay for determining the levels of neutralizing antibody against WNV NY99 in mice sera. This method is recommended by WHO for testing the potency of Japanese encephalitis vaccine. The cut-off value for testing serum seroprotection is 1 log_10_ (a ten-fold dilution of serum that reduces plaque formation by 50 % is sufficient for protection against viral challenge) [32]. In an earlier study, mice immunized actively or passively that possessed WNV-neutralizing antibodies higher than 1 log_10_ were protected against the lethal WNV challenge [33]. In our experiments, the neutralizing antibody titer of rLa-WNV-PrM/E-immunized mice reached up to 1.3 log_10_ after the first dose. After administration of the second dose, the neutralizing antibody titer was significantly boosted (~2.2 log_10_), implying that rLa-WNV-PrM/E confers robust protection against lethal WNV infection in mice. In sera of mice, high levels of anti-WNV E IgG were elicited after the first dose, which were significantly boosted after the second dose. IgG findings were in accordance with the neutralizing antibody pattern, indicating a linear correlation in rLa-WNV-PrM/E-immunized animals. T-cell responses are additionally critical in controlling WNV infection. CD4+ T-cells play a dominant protective role in viral clearance via facilitating antibody responses and sustaining WNV-specific CD8+ T-cell responses in the central nervous system (CNS) [34]. The efficacy of CD8+ T cells in controlling WNV infection has also been characterized. An earlier study showed that while neutralizing antibodies play a central role in terminating WNV viremia, CD8+ T-cells are essential for preventing sustained WNV infection in peripheral and CNS compartments [35]. Live vector vaccines have a significant advantage in that they effectively elicit cellular responses [36–38]. In our experiments, rLa-WNV-PrM/E induced high levels of WNV-specific CD4+ and CD8+ T-cell responses. Although a challenge study was not conducted at this time due to the unavailability of the BSL-3 laboratory, given the neutralizing antibody and T-cell response results, we presume that the rLa-WNV-PrM/E could confer protection in mice. Intramuscularly immunized horses produced WNV specific IgG following the first dose, which was significantly boosted after the second dose. Considering the close association between IgG and neutralizing antibody levels, we propose that horses acquire protective immunity after vaccination. Further neutralization assays and challenge studies are required to confirm the efficacy of the vaccine in horses.

Vaccination of sensitive hosts not only protects the animal itself but also prevents transmission of WNV from animals to humans. Several veterinary WNV vaccines are currently available, including inactivated whole virus vaccine [5, 39, 40], DNA vaccines [41–43], recombinant canarypox-vectored vaccine [6, 44] and recombinant Yellow Fever 17D vaccine [45, 46]. Notably, canarypox-vectored WNV vaccine has been shown to effectively elicit WNV-specific neutralizing antibodies and confer protection in horses, geese, cats and dogs against lethal WNV challenge [6, 44, 47, 48]. These vaccines require delivery via intramuscular inoculation. The current study demonstrated that rLa-WNV-PrM/E is immunogenic in not only mice, horses and poultry when administered intramuscularly, but also in poultry upon delivery via intranasal or oral inoculation. Based on the collective findings, we propose that rLa-WNV-PrM/E is a promising veterinary candidate vaccine for multiple mammalian and avian species that can be delivered via flexible inoculation routes.

Domestic poultry, such as chickens, ducks and geese, are susceptible to WNV and develop clinical signs and viremia (usually sufficient to infect mosquitoes), thus contributing to WNV transmission. Chickens are widely used as sentinel animals in the early warning of WNV prevalence [2, 49–52]. Several studies have additionally provided evidence of the susceptibility of domestic or captive ducks to WNV [3, 53, 54]. Ducks develop high-titer viremia in blood and are capable of shedding virus orally [55]. Geese are also susceptible to WNV, especially young geese, and develop a variety of neurological signs, often resulting in a significant number of deaths [4, 56, 57]. Geese represent another experimental animal model for WNV vaccine evaluation [58–60]. Notably, WNV human infection and isolation of the virus has recently been reported in mosquitoes in the Xinjiang Uygur Autonomous Region in Northwest China [61, 62]. China has an estimated 12 billion or more poultry, including four billion ducks and geese. Many domestic birds, especially ducks and geese, are raised in backyards and open ranges under poor biosecurity conditions and live in high-density groups close to large human populations. Since these birds may serve as important amplifying hosts of WNV, control of WNV circulation in birds is important for public health. For economic reasons, poultry farmers are generally unwilling to pay additional vaccine and labor costs for vaccination solely against WNV. As NDV is one of the most lethal and economically important pathogens for poultry (at least chickens and geese), farmers use live vaccines, such as LaSota strain, to protect against infection. In our study, most domestic poultry showed NDV HI antibody titters higher than 4 log_2_ after vaccination, irrespective of the delivery route (Figs. 5b, 6b and 7b). A HI antibody titer higher than 3 log_2_ is usually sufficient to protect poultry from lethal challenge of NDV. In this scenario, farmers will not need to pay additional vaccine and labor costs to protect against WNV infection by using rLa-WNV-PrM/E instead of NDV live vaccine for routine vaccination. Moreover, oral and intranasal immunization routes are more convenient than intramuscular immunization for poultry as well as water fowl and migratory and resident wild birds.

## Conclusions

In summary, rLa-WNV-PrM/E vaccination in susceptible animals is important for protection against WNV infection. Our findings demonstrate that rLa-WNV-PrM/E delivered via multiple immunization routes is immunogenic in both mammals and poultry.
